# Potential prebiotic effect of two Atlantic whole brown seaweeds, *Saccharina japonica* and *Undaria pinnatifida*, using *in vitro* simulation of distal colonic fermentation

**DOI:** 10.3389/fnut.2023.1170392

**Published:** 2023-04-14

**Authors:** Aroa Lopez-Santamarina, Laura Sinisterra-Loaiza, Alicia Mondragón-Portocarrero, Jaime Ortiz-Viedma, Alejandra Cardelle-Cobas, Carlos Manuel Franco Abuín, Alberto Cepeda

**Affiliations:** ^1^Laboratorio de Higiene, Inspección y Control de Alimentos (LHICA), Departamento de Química Analítica, Nutrición y Bromatología, Facultad de veterinaria, Universidade de Santiago de Compostela, Lugo, Spain; ^2^Departamento de Ciencia de los Alimentos y Tecnología Química, Facultad de Ciencias Químicas y Farmacéuticas, Universidad de Chile, Santiago, Chile

**Keywords:** gut microbiota, *in vitro*, fermentation, seaweed, *Bifidobacterium*, 16S rRNA

## Abstract

Two brown seaweeds (*Saccharina japonica* and *Undaria pinnatifida*) were characterized in terms of their nutritional and mineral composition, as well as their potential to modify the human gut microbiota. Nutritional analysis of these seaweeds showed that they comply with the criteria set out in European legislation to be labeled “low fat,” “low sugar,” and “high fiber.” Mineral content analysis showed that 100 g of seaweed provided more than 100% of the daily Ca requirements, as well as 33–42% of Fe, 10–17% of Cu, and 14–17% of Zn requirements. An *in vitro* human digest simulator system was used to analyze the effect of each seaweed on the human colonic microbiota. The gut microbiota was characterized by 16S rRNA amplicon sequencing and short-chain fatty-acid analysis. Seaweed digestion and fermentation showed beneficial effects, such as a decrease in the phylum Firmicutes and an increase in *the phyla* Bacteroidetes and Actinobacteria. At the species level, seaweed fermentation increased the proportion of beneficial bacteria such as *Parabacteroides distasonis* and *Bifidobacterium*. Regarding of metabolic pathways, no significant differences were found between the two seaweeds, but there were significant differences concerning to the baseline. An increase in short-chain fatty-acid content was observed for both seaweeds with respect to the negative control, especially for acetic acid. Given of the obtained results, *S. japonica* and *U. pinnatifida* intake are promising and could open new opportunities for research and application in the fields of nutrition and human health.

## 1. Introduction

Human consumption of seaweed has been occurring for millennia in Asian populations (1). Additionally, in recent decades, seaweed consumption has increased in Western populations due to its association with human health benefits. Seaweeds are of interest as food for humans due to their content of micro-and macronutrients; trace elements such as Zn, Ca, or I; lipids in the form of polyunsaturated fatty acids and phytosterols; and proteinogenic amino acids and peptides; and natural pigments such as chlorophylls, phycobiliproteins, and carotenoids (2). However, the seaweed component that has received the most attention in recent years is dietary fiber, mostly composed of nondigestible polysaccharides that display numerous health-promoting properties, such as antitumoral, antiviral, or antioxidant activities (2). In global terms, the consumption of dietary fiber in adequate amounts confers numerous human health benefits (3). For this reason, an increase in dietary fiber has been persistently recommended in Western societies since the 1970s to reach an intake of 25–35 g/day in adults (4).

Currently, most of the nondigestible polysaccharides in the Western diet are derived from the cell walls of terrestrial plants (5). However, Western inhabitants average dietary fiber intake remains continuously below the recommendations, partly because part of the population is reluctant to consume vegetables in the recommended amounts. For this reason, an increase in seaweed consumption in the Western population could be an interesting alternative to terrestrial vegetables as a source of dietary fiber (5). Additionally, due to continuous intense worldwide population growth, resources needed for land-based agriculture, such as arable land and water for irrigation, are becoming increasingly scarce (6). Consequently, the demand for foods from other sources is expected to increase in the coming years. Seaweed cultivation has numerous advantages over terrestrial plants, as its growth rate is high, and it does not require arable land, fresh water, or fertilizers (7). Increasing seaweed cultivation and food production *per se* would bring environmental benefits, such as CO_2_ fixation (8).

The consumption of dietary fiber has human health benefits that are directly related to the digestive system. The digestive tract is the main area of exchange and communication with the external environment in the human body. The gastrointestinal mucosa in an adult human covers an area of 300–400 m^2^ (considering the entire surface, with the villi deployed), and thanks to its structure and functions, it specifically recognizes substances that pass through the gastrointestinal tract (9). These functions dependent not only on the structure of the digestive tract itself but also on the presence and activity of microbial communities that colonize the gut and play an important role in the homeostasis and environmental balance of the individual (10). As seaweed is a food with a high content of dietary fiber (in some seaweed species, dietary fiber can represent more than 70% of total dry weight), its consumption can contribute to the improvement of these functions in human health.

Currently, most studies investigating the potential effects of seaweed on the gut microbiota (GM) using *in vitro* or *in vivo* animal models have been performed using polysaccharides extracted from seaweeds, not considering the effects that other components of seaweed may have on the GM. The most studied group in terms of their prebiotic activity is brown seaweed, as it is the group of seaweed that has shown the most beneficial effects in this regard (5). Most of these studies have demonstrated their efficacy as prebiotic ingredients (11–14). However, some minor components of foods can also exert an important effect on GM composition and functionality (15), among which some antioxidant compounds, such as polyphenols, have been particularly studied (15). Consequently, because of its content of minor compounds such as pigments, minerals, peptides, or fatty compounds, seaweed consumption can exert effects on the human GM in addition to those derived exclusively from its content of nondigestible polysaccharides (5, 16).

Previous studies with polysaccharides extracted from *Saccharina japonica* and *Undaria pinnatifida* have already reported prebiotic effects and improvement of GM composition in mice (17, 18). Thus, taking into account that seaweed contains minor compounds that can improve GM composition and functionality, the aim of this work was to investigate the potential prebiotic effect on the human GM using an *in vitro* simulator of two common brown seaweeds, “kombu” (*S. japonica*) and “wakame” (*U. pinnatifida*), obtained from the Galician coast (NW Spain), a marine area with high seaweed production.

## 2. Materials and methods

### 2.1. Seaweeds

Both *S. japonica* and *U. pinnatifida* were obtained dehydrated from Portomuiños (Cerceda, A Coruña, Spain). A total of 250 g of each seaweed was crushed and freeze-dried. Afterward, seaweeds were stored at room temperature in a desiccator until analysis and *in vitro* digestion, for a period of a week after reception.

### 2.2. Nutritional and mineral composition of *Saccharina japonica* and *Undaria pinnatifida*

The nutritional composition of *S. japonica* and *U. pinnatifida* was determined prior to and after *in vitro* digestion. Nutritional composition analysis was carried out according to methodologies established by the Association of Official Analytical Chemists (19). The moisture content (g/100 g of product) was determined by drying at 100–105°C in an oven. The protein content was determined by measuring the nitrogen content (g/100 g of dry matter) using the Kjeldahl method. The fat content (g/100 g of dry matter) was determined by Soxhlet extraction. The dietary fiber content (g/100 g of dry matter) was determined by the enzymatic–gravimetric method using a Megazyme^®^ total dietary fiber assay kit (Megazyme, Wicklow, Ireland). Ash content (g/100 g of dry matter) was determined by incineration at 500°C in a muffle furnace. The sodium content (mg/100 g of dry matter) was determined by atomic absorption spectrometry using an Agilent 5,900 spectrometer (Agilent Technologies, Santa Clara, California, USA). The carbohydrate (g/100 g of dry matter) and caloric contents (kcal/100 g of product) were determined through calculations.

Regarding mineral content, seaweeds were analyzed for ^75^As, ^43^Ca, ^112^Cd, ^63^Cu, ^56^Fe, ^202^Hg, ^127^I, ^208^Pb, and ^66^Zn (mg/kg) by inductively coupled plasma–mass spectrometry (Agilent 7,700x, Agilent Technologies, Santa Clara, CA, USA). Sample blanks were prepared in the laboratory in a similar manner to the seaweed samples. All nutritional and mineral composition determinations were performed in triplicate before and after *in vitro* digestion.

### 2.3. *In vitro* simulation of oral, gastric, and small intestinal digestion

*In vitro* simulation of oral, gastric, and small intestinal digestion was performed according to the INFOGEST protocol (20), adapted to use 10 g of each seaweed. All chemicals required were obtained from Sigma–Aldrich (St. Louis, MO, USA).

At the end of the *in vitro* digestion process, the enzymatic reactions were stopped by cooling the digested seaweed solutions. Absorption in the small intestine was then simulated using membrane dialysis (1,000 Da molecular-weight cutoff, Spectra/Por^®^, Waltham, MA, USA) with distilled water at 4°C for 2 days with continuous stirring. This process allows the molecules in solution to be separated by the difference in their diffusion rates through the semipermeable membrane. The contents of the membrane were then frozen at −18°C for subsequent freeze-drying. The freeze-drying process was performed by using a vacuum freeze-dryer (Labconco™ 77,560-LYPM-LOCK6) under a vacuum pressure of ≤140 × 10^−3^ Mbar and a condenser temperature of −46°C. All processes were performed in triplicate.

### 2.4. Volunteers and stool samples

Three healthy human volunteers (one male and two females, aged 32–50 years) who participated in a trial authorized by the Galician Bioethics Committee (trial 270/2018) donated their feces for this trial. Intake of antibiotics or pharmaceutical pre/pro/postbiotics as well as suffering gastrointestinal disorders in the 6 months prior to sampling were used as exclusion criteria. Volunteers signed an informed consent form informing them of how their samples would be used, the study’s compliance with the Declaration of Helsinki, and the Spanish personal data protection law. Donors brought 10–30 g of feces in sterile containers and delivered them to the laboratory no later than 2 h after collection. These stool samples, in the laboratory, were diluted 1:10 with phosphate buffered saline (PBS; 0.1 M, pH 7.0) and then homogenized by using a paddle homogenizer (MIX2, AES, Combourg, France) for 5 min. Diluted feces of each volunteer were stored in sterile vials and frozen at −20°C until use.

### 2.5. *In vitro* human colonic simulation

The *in vitro* human colonic simulation was performed following the method described by Cardelle-Cobas et al. (21). Briefly, a sterilized fermentation vessel containing a basal culture medium without any source of carbon was used to simulate human distal colonic fermentation. In addition to *S. japonica* and *U. pinnatifida* sample fermentation, a negative control without a carbon source was performed for each voluntary assay.

The conditions of the human distal colon were simulated in terms of anaerobic atmosphere, temperature, and pH. An anaerobic atmosphere was achieved by a continuous supply of pure grade N_2_ (Nippon Gases, Madrid, Spain) through a 0.2-μm polytetrafluorethylene filter (Sartorius Stedim Biotech GmbH, Gottingen, Germany). The human internal body temperature (37°C) was simulated by continuous recirculation. The pH was adjusted to 6.8, simulating a eubiotic colonic pH, by the addition of appropriate amounts of 1 M NaOH or HCl, controlled by continuous measurement with a pH regulator (Hanna Instruments, Eibar, Spain).

Under aseptic conditions, 200 mL of sterile nutrient basal medium was added to each fermentation vessel (21), the pH was adjusted to pH 6.8, and the culture was left overnight in a stream of O_2_-free N_2_ with stirring. Next, the sterilized substrates (*S. japonica* or *U. pinnatifida*) were dissolved in 52 mL of the same autoclaved medium and added to the vessels to achieve a final concentration of 1% (w/v). Finally, the vessels were inoculated with 10% (v/v) (28 mL) of the previously prepared diluted feces. All chemical compounds required were obtained from Sigma–Aldrich, Merck (Darmstadt, Germany), or Panreac (Barcelona, Spain).

Sample aliquots (5 mL) were taken from each vessel after 0, 10, 24, and 48 h of fermentation for bacterial DNA extraction and used for ribosomal RNA (rRNA) amplicon sequencing.

### 2.6. Bacterial DNA extraction from fermentation samples and 16S ribosomal RNA amplicon sequencing

Bacterial DNA was extracted from the fermented samples with the DNA Realpure Spin Food-Stool Kit® (Real, Durviz S. L, Valencia, Spain) according to the protocol established by the manufacturer for fecal samples. A total of 1.2 mL of sample obtained from the fermentation vessels was centrifuged at 6,100 *g* to obtain a pellet, which was recovered and used for DNA extraction. Extracted DNA was then quantified using a Qubit™ 4 fluorometer (Invitrogen, Thermo Fisher Scientific, Carlsbad, CA, USA) and the DNA HS Assay Kit (Invitrogen, Thermo Fisher Scientific, Eugene, OR, USA) according to the manufacturer’s instructions. After quantification, DNA samples were stored frozen at −20°C until further analysis.

For 16S rRNA amplicon sequencing, the same procedure was followed as previously reported in a previous work (22).

### 2.7. Short-chain fatty-acid analysis

Short-chain fatty-acid (SCFA) analysis was performed according to the protocol described by Gullón et al. (23). One-milliliter samples obtained after 0, 10, 24, and 48 h of fermentation were centrifuged for 7 min at 6100 *g*. The supernatants were removed and filtered through 0.2-μm cellulose acetate membranes (Phenomenex, California, USA). Then, 20 μL of each sample was injected into an Aminex HPX-87H column (LC Column 300 × 7.8 mm; Bio-Rad, Hercules, California, USA) operating at 50°C. The mobile phase, 3 mM sulfuric acid, was flashed to the column at a flow rate of 0.6 mL/min in isocratic mode, and the temperature of the column was constant during the whole run at 50°C. Analysis was performed with an HPLC-PDA system from Agilent (Waldbronn, Germany) consisting of a binary pump, a degasser, an autosampler, and a column heater coupled to a detector (Infinity 1260 II Diode Array Detector HS; Agilent, Waldbronn, Germany). Organic acids (lactic, acetic, butyric, propionic, isobutyric, valeric, and isovaleric acids) were obtained from Sigma (Poole, Dorset, United Kingdom). The SCFA content was determined by comparing their retention times with those of standards and quantified employing the regression formula obtained by plotting different concentrations of the fatty acid against its corresponding area.

### 2.8. Statistical and bioinformatic analysis

The results of nutritional composition and mineral content before and after digestion simulation were subjected to statistical comparison by paired Student’s *t-*test. Differences between different SFCAs at different fermentation times were compared by analysis of variance (ANOVA) and a *post hoc* Tukey test. SPSS® for Windows v.22 (SPSS Inc., Chicago, IL, USA) was used for these analyses. In all cases, differences were considered significant at *p* < 0.05.

For 16S rRNA amplicon sequencing analysis, raw sequencing reads were obtained from Torrent Suite software (v.5.12.2.) as fastq files, which were downloaded from the plugin “Metagenomics.” The fastq files were processed with QIIME 2 software v. 2021.8 (24) and subsequently with Microbiome Analyst.^1^ To produce amplicon sequence variants, the DADA2 method was used for quality filtration (Q score > 10), trimming, denoising, and dereplication. Samples with features (taxa) with a total abundance (summed across all samples) of <20 were removed and were normalized by rarefaction. Taxonomy was assigned to ASVs by using the q2-feature-classifier classify-sklearn naïve Bayes taxonomy classifier against the Greengenes 13_8 99% operational taxonomic unit (OTU) reference sequences. Alpha (α)-diversity statistical analysis was processed by MicrobiomeAnalyst based on OTU data using ANOVA (for three or more groups).

To understand the number (richness) and/or distribution (evenness) of OTUs within a sample, two different alpha diversity indices were estimated: (i) the Chao1 index, an abundance-based index of OTUs richness (25, 26), and (ii) the Shannon index, an index of both OTUs richness and evenness (27). *β*-diversity, considered individual-level information between samples (28), was measured by the Bray–Curtis index, measuring the distance matrix considering the relative abundances of OTUs. Associations between-sample (*β*-diversity) of the GM were examined by permutational multivariate analysis of variance.

Metagenome functional content from marker gene surveys and full genomes was predicted by the PICRUSt online Galaxy version on the Huttenhower Lab (v1.0.0) server. Functional metagenomes were categorized based on the Kyoto Encyclopedia of Genes and Genomes (KEGG) pathway database at hierarchy level 3.

Welch’s *t-*tests with Bonferroni correction were used to determine significant differences in the relative abundance of 20 selected KEGG pathways using STAMP software (v 2.1.3). In addition, differences in the relative abundance of the most common species were determined by using a G-test (with Yates’ correction) + Fisher’s exact test with Bonferroni correction.

## 3. Results and discussion

### 3.1. Nutritional and mineral composition of *Saccharina japonica* and *Undaria pinnatifida*

The nutritional and mineral compositions of *S. japonica* and *U. pinnatifida* are shown in Table 1. Although the nutritional content of seaweeds can vary due to multiple external factors, such as seaweed species, sea salinity, seasonality, temperature, or sunlight intensity (29), the results of this work, expressed on a dry matter basis, agree with those obtained previously by Fernandez-Segovia et al. (30) on the same species of seaweeds. This study estimated a protein content of 7.9% for *S. japonica*, compared with the approximately 8% reported by Fernandez-Segovia et al. (30). For *U. pinnatifida,* the protein percentages were 16.6% vs. 19.0%. For the fat content, in the case of *S. japonica,* the results were similar to those reported by Fernandez-Segovia et al. (30) (<1% fat content), but in the case of *U. pinnatifida*, in the current work, the fat content reached 3%, whereas Fernandez-Segovia et al. (30) reported a fat content below 1%. Although the lipid content was low, the two seaweed species investigated contained mainly polyunsaturated fatty acids (PUFAs), as reported by Peinado et al. (31). This low lipid content, together with its fatty-acid profile, is an advantage of including seaweed in the human diet. In this sense, a study conducted on dry pasta made with semolina and seaweed mixtures showed that the addition of *U. pinnatifida* increased the *ω*-3 fatty-acid content of pasta (30). For dietary fiber, the results were also similar to those reported by Fernandez-Segovia et al. (30) (37.7% vs. 44.0% for *S. japonica* and 34.4% vs. 39.0% for *U. pinnatifida*, respectively), in agreement with previous work carried out on another brown seaweed species (22). The ash content was in close agreement with Fernández-Segovia et al. (30) (29.5% vs. 30.0%). Regarding the caloric content, the results of this work coincide with those obtained in a previous work carried out on *Himanthalia elongata* (22). According to their nutritional composition, *S. japonica* and *U. pinnatifida* could be marketed in the European Union using the claims “low fat content” (<3 g of fat/100 g product), “low sugar content” (<5 g sugar/100 g product), and “high content of dietary fiber” (>3 g of dietary fiber/100 g product), as established by European Regulation (32). These claims would encourage the purchase of these foods and, therefore, their consumption.

**Table 1:** Comparison of the nutritional composition (g/100 g) and mineral content (mg/kg) of kombu (*Saccharina japonica*) and wakame (*Undaria pinnatifida*) raw and after *in vitro* upper intestinal digestion.

Nutritional composition (% DW matter)	*Saccharina japonica*		*Undaria pinnatifida*	
	Raw	After upper *In vitro* digestion	Raw	After upper *In vitro* digestion
Moisture	8.17^a^ ± 0.30	<0.01^b^	9.29^a^ ± 0.22	<0.01^b^
Protein	7.90^b^ ± 0.04	13.14^a^ ± 0.26	16.64 ± 0.68	17.88 ± 0.12
Fat	<0.5	<0.5	3.00a ± 0.02	0.78b ± 0.01
Carbohydrates	16.28^a^ ± 0.39	10.60^b^ ± 1.45	8.43 ± 0.32	9.66 ± 0.35
Sugars	<0.5	<0.5	<0.5	<0.5
Dietary fiber	37.72^b^ ± 0.45	62.24^a^ ± 0.39	34.44^b^ ± 0.29	59.76^a^ ± 0.45
Ash	29.53^a^ ± 0.04	13.29^b^ ± 0.16	28.24^a^ ± 0.59	11.92^b^ ± 0.78
Caloric content (kcal/ 100 g)	176.62^b^ ± 0.78	226.01^a^ ± 0.32	198.08^b^ ± 0.23	236.70^a^ ± 1.23
Minerals (mg/kg)				
Ca	16,100.62^b^ ± 823.93	33,877.98^a^ ± 705.62	17,705.17 ± 939.86	32,752.79 ± 6872.0
Fe	47.40^b^ ± 2.18	73.60^a^ ± 7.10	69.65 ± 6.93	130.00 ± 43.92
Cu	1.78^b^ ± 0.11	4.09^a^ ± 0.031	1.26 ± 0.037	2.50 ± 0.85
Zn	17.69^b^ ± 0.49	52.41^a^ ± 4.05	16.91 ± 0.85	44.86 ± 12.45
As	1.83 ± 0.34	4.34 ± 0.21	2.13 ± 0.35	5.74 ± 3.22
Cd	0.07 ± 0.02	0.20 ± 0.10	0.19 ± 0.08	0.77 ± 0.59
Hg	<0.001	<0.001	<0.001	<0.001
Pb	0.19 ± 0.06	0.22 ± 0.03	0.11 ± 0.07	0.33 ± 0.07
I	2,355.2^a^ ± 216.76	14.59^b^ ± 3.11	128.91^a^ ± 8.76	7.13^b^ ± 0.78

Different letters indicate significant differences (*p* < 0.05) in nutritional content before and after digestion of each seaweed. The standard deviation is expressed next to each average.

Food digestibility can be affected by gastrointestinal luminal alterations (such as the composition of digestive fluids or intestinal pH). After upper *in vitro* digestion, an increase in protein content was found, which reached statistically significant levels in the case of *S. japonica*. The lipid content decreased only in the case of *U. pinnatifida*, which agrees with a previous study carried out with another brown seaweed (*H. elongata*) (22). It also increased the dietary fiber content by 50%, as well as the calorie content. By contrast, the carbohydrate content (only in the case of *S. japonica*) and the ash content decreased. After upper digestion, protein and dietary fiber become the main components of the two seaweeds and therefore act to a greater extent at the colonic level on the intestinal microbiota.

Table 1 also shows the content of minerals and heavy metals of *S. japonica* and *U. pinnatifida* before and after *in vitro* digestion simulating the upper gastrointestinal tract. The heavy metal content of raw dried seaweed was similar to that obtained in another study on European seaweed (33).

With respect to the maximum content limits for minerals, in the European Union (EU), only a specific limit for iodine in seaweed has been established (20 mg I/kg dry seaweed) (34). The iodine content is one of the main risk factors for seaweed overdosage, but seaweeds are usually hydrated prior to consumption, which increases their volume. The moisture content of hydrated *S. japonica* is 89%, which reduces the I content by a factor of 8. In addition, Cascais et al. (35) showed that the blanching technique (80°C for 120 s) reduced the I content in brown seaweed from 4,605 to 293 mg iodine/kg/dw.

The mineral content results show that in both raw *S. japonica* and *U. pinnatifida*, the contents are below maximum limits established by Regulation (EC) 1881/2006 (36) of the European Commission specifically for seaweeds for Ca, Fe, Cu, and Zn or those established by the European Food Safety Authority (EFSA) for Pb, Hg (37), and Cd (38). These data agree with those obtained in previous similar work with *H. elongata* (22).

In addition, it is important to note that some minerals, such as Ca, Fe, Cu, or Zn, are the most essential trace elements and minerals and are necessary for human growth, enzymatic reactions, and metabolic activities (39), as well as for the metabolism of the GM (40). Usually, the consumption of Ca, Fe, Cu, and Zn is below the recommended levels (41, 42). The results of this work show that 100 g of *S. japonica* provided 202, 33, 17, and 17% of the minimum daily requirements for Ca, Fe, Cu, and Zn, respectively (43). In the case of U*. pinnatifida,* the supply per 100 g of seaweed was similar (191% of Ca, 42% of Fe, 10% of Cu, and 14% of Zn).

After digestion in the upper intestinal tract, the mineral content increased significantly in the case of the Ca, Fe, Cu, and Zn contents of *S. japonica*, whereas this did not occur in the case of *U. pinnatifida*. By contrast, the I content decreased significantly after *in vitro* digestion in the upper intestinal tract in both seaweeds. It has been reported that in *S. japonica,* there is a higher percentage of I as I^−^, which can pass through the dialysis membrane, which would explain the higher dualizability of iodine in *S. japonica* using this *in vitro* approach (44). The explanation for the observed changes related to the concentration of minerals may be that in addition to their simple form, minerals can also be complexed with organic compounds such as phenolic compounds and proteins, which can affect their bioavailability (45). In some cases, minerals are concentrated in samples after digestion and dialysis. This may be because a membrane with a very small pore size (1 kDa) was used in this work, and it is possible that some minerals may not be able to pass through it. A previous study found similar results when investigating the intestinal bioavailability of the seaweed *U. pinnatifida* (44).

### 3.2. Amplicon 16S rRNA sequencing

**Figure 1:**
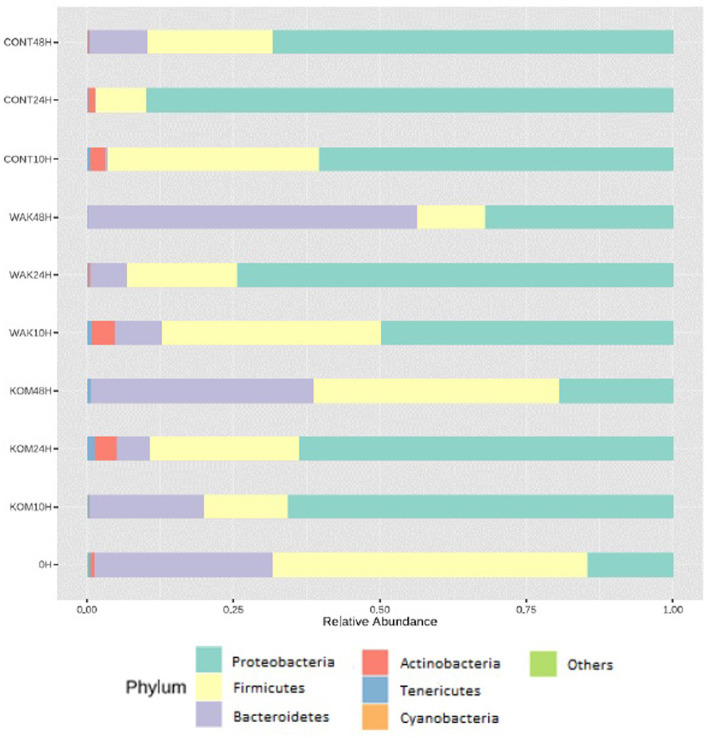
Relative abundance of different bacterial phyla. Bacterial composition (relative abundance, %) determined using 16S rRNA amplicon sequencing at the phylum. The y axis shows the different substrates evaluated at the different assay times (10, 24, and 48 h). 0 h indicates the bacterial composition before substrate addition. CONT (negative control, without substrate); KOM (*S. japonica*, kombu); WAK (*U. pinnatifida*, wakame).

Figure 1 shows that both *S. japonica* and *U. pinnatifida* modified the GM compared to controls. At time 0, the phylum Firmicutes predominates, followed by Bacteroidetes, with the proportion of Proteobacteria being lower. These results are consistent with recent similar studies. For example, Vázquez-Rodríguez et al. (13) studied the effect of polysaccharide fractions of brown seaweed on human GM under the same conditions. They found that the relative abundance of Proteobacteria increased with fermentation time for negative controls. An increase in the proportion of Proteobacteria also occurred in samples supplemented with both *S. japonica* and *U. pinnatifida*, but the increase was smaller in the case of negative controls. Deng et al. (46) also showed an increase in the relative abundance of Proteobacteria by supplementation with dietary fiber extracted from *S. japonica*. By contrast, at 48 h, the Bacteroidetes phylum became predominant, whereas in the negative control, a significant decrease was observed. Similar results were observed in a previous study using inulin (common prebiotic) (22), in which the Bacteroidetes phylum predominated at 48 h, as observed in the cases of both *S. japonica* and *U. pinnatifida*. Jiang et al. (17) showed an increased proportion of the Bacteroidetes phylum with the administration of sulfated polysaccharides from *U. pinnatifida*. It is important to note that in the case of *S. japonica* at 24 h and *U. pinnatifida* at 10 h, a significant increase in the abundance of the Actinobacteria phylum was observed. This could be of interest, as this phylum includes the species *Bifidobacterium*, which exerts beneficial effects on the host’s health and is often used as a probiotic agent (47).

**Figure 2:**
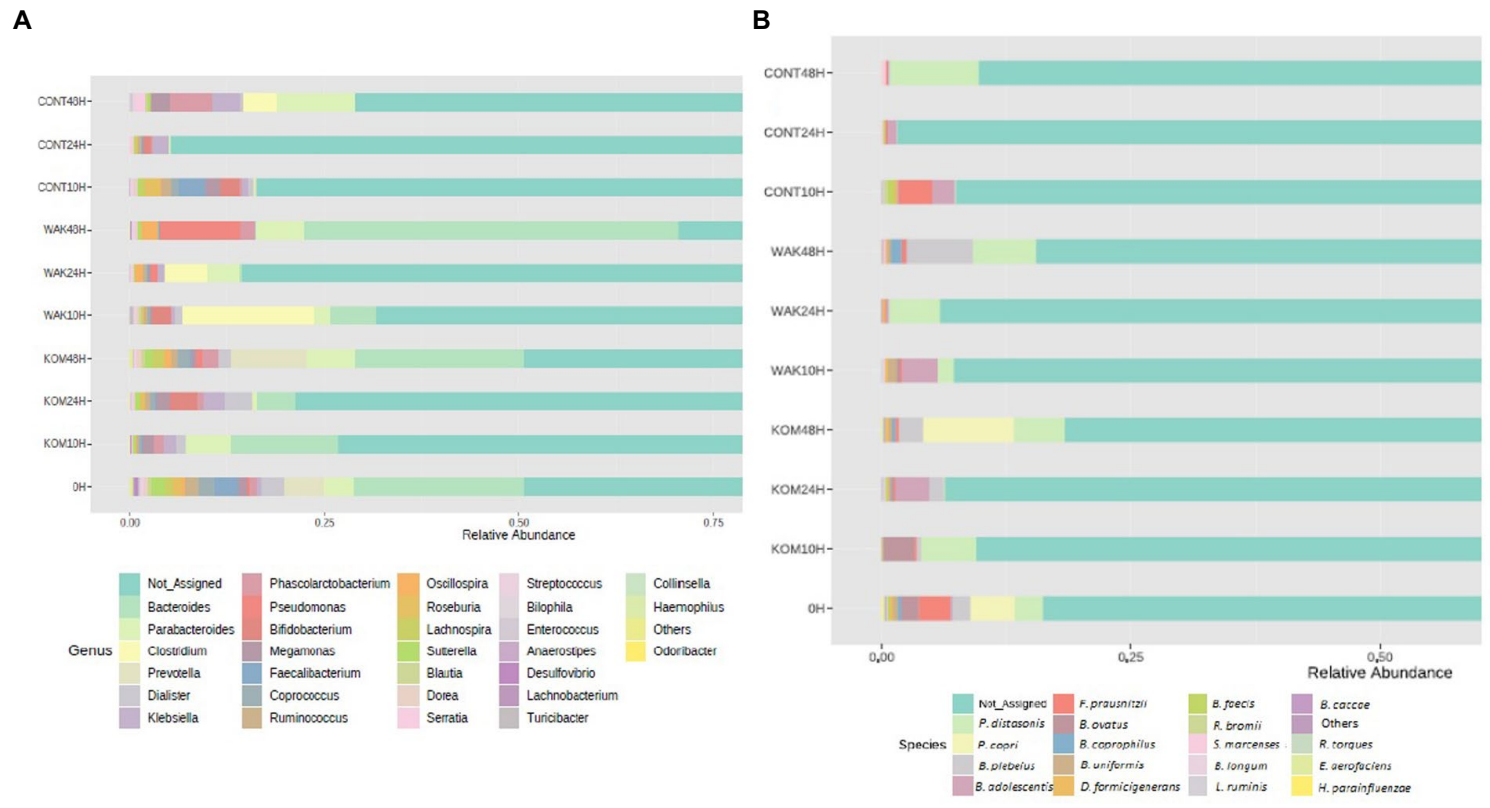
Relative abundance of most representative genus and species identified. Bacterial composition (relative abundance, %) determined using 16S rRNA amplicon sequencing at the genus **(A)** and specie **(B)** levels. The y axis shows the different substrates evaluated at the different assay times (10, 24, and 48 h). 0 h indicates the bacterial composition before substrate addition. CONT (negative control, without substrate); KOM (*S. japonica*, kombu); WAK (*U. pinnatifida*, wakame).

Regarding bacterial genera (Figure 2A), the *Bacteroides* genus showed the highest relative abundance, followed by *Parabacteroides*. A previous work carried out with polysaccharides extracted from *S. japonica* showed similar results regarding *Bacteroides* (18). The same results were obtained in another study using polysaccharides extracted from *U. pinnatifi*da (17). Previous studies documented a few examples in which bacteria belonging to *Bacteroides* possess genes for degrading seaweed-derived porphyrin agarose, alginate, and laminarin (48).

In terms of bacterial species (Figure 2B), one of the most representatives is *Parabacteroides distasonis*, as in the case of a similar study carried out with inulin (22). Another bacterial species, *Prevotella copri*, increases after 48 h of fermentation in *S. japonica*. This result is notable because *P. copri* has been shown to contribute to the improvement of glucose metabolism in mice, potentially through succinate-activated intestinal gluconeogenesis (49). In the same seaweed, a significant increase in *Bifidobacterium adolescentis* was observed at 24 h in the case of *S. japonica* and at 10 h in the case of *U. pinnatifida*. This finding has a positive effect, as different *Bifidobacterium* species, *including B. adolescentis*, are widely marketed as probiotics (50).

**Figure 3:**
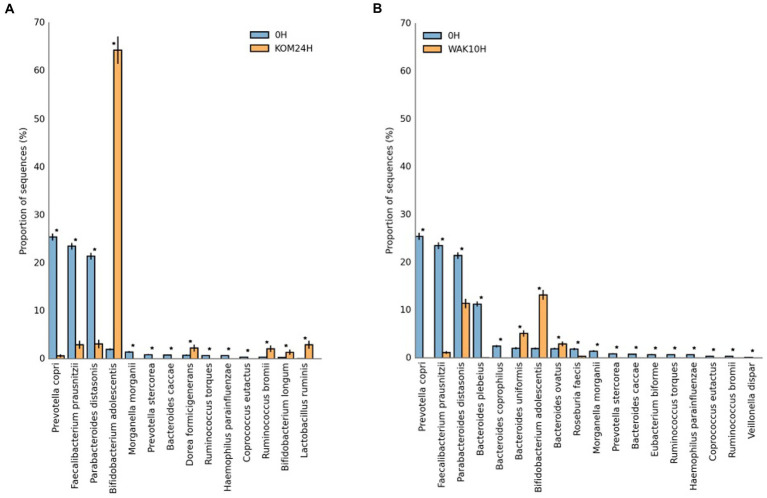
Statistical differences for the more abundant identified bacterial species in *S. japonica*-added samples at 24 h **(A)** and *U. pinnatifida*-added samples at 10 h **(B)** of fermentation with respect to baseline. KOM (*S. japonica*, kombu); WAK (*U. pinnatifida*, wakame). *Indicates significant differences.

Many of the other species that show a significant increase during seaweed fermentation belong to the *Bacteroides* genera (especially *B. plebeius, B. ovatus*, *B. coprophilus*, and *B. uniformis*). Other experiments revealed that some Bacteroidales members were positively correlated with the bioactivity of polysaccharides extracted from *U. pinnatifida*. It was shown that *B. ovatus* could grow by fermenting these polysaccharides (17). It has been reported that gavage with *B. ovatus* significantly lowered body weight gain and fat accumulation in high-fat diet-fed mice (51). Furthermore, *B. ovatus* demonstrated a potentially beneficial role in the setting of colitis. Because the *Bacteroides* genus specializes as primary degraders in the metabolism of complex carbohydrates, they can gain a competitive advantage in the degradation of polysaccharides from both seaweeds. This fact was previously reported in another study using polysaccharides extracted from brown seaweeds (52). Another study carried out with polysaccharides extracted from *S. japonica* showed similar results, wherein the relative proportion of six species of Bacteroidetes increased after fermentation (18). The most important changes at the species level occur at 24 h of fermentation in the case of *S. japonica* and at 10 h in the case of *U. pinnatifida*. This can be seen depicted in Figure 3, which shows statistical differences for the more abundant identified bacterial species in *S. japonica-*added samples at 24 h (3A) and *U. pinnatifida*-added samples at 10 h (3B) of fermentation with respect to baseline. In the case of *S. japonica* 24 h after fermentation, a noticeable increase of *B. adolescentis*, which is considered probiotic (15), can be seen. The same occurs, although to a lesser extent, with other bacteria that have demonstrated their probiotic effect, such as *Ruminococcus bromii*, *Bifidobacterium longum* and *Lactobacillus ruminis*. As far as *U. pinnatifida* is concerned, after 10 h of fermentation, a growth of *B. adolescentis* can also be seen. Although the data have not been shown, it is also important to note that there are no significant species differences between *S. japonica* 24 h and *U. pinnatifida* 10 h. Previous work with polysaccharides extracted from these brown seaweeds has shown similar results in terms of growth of *Bifidobacterium* and *Lactobacillus* species (52). These results are indicative of the possible prebiotic effect that both seaweeds may have on the human gut microbiota.

**Figure 4:**
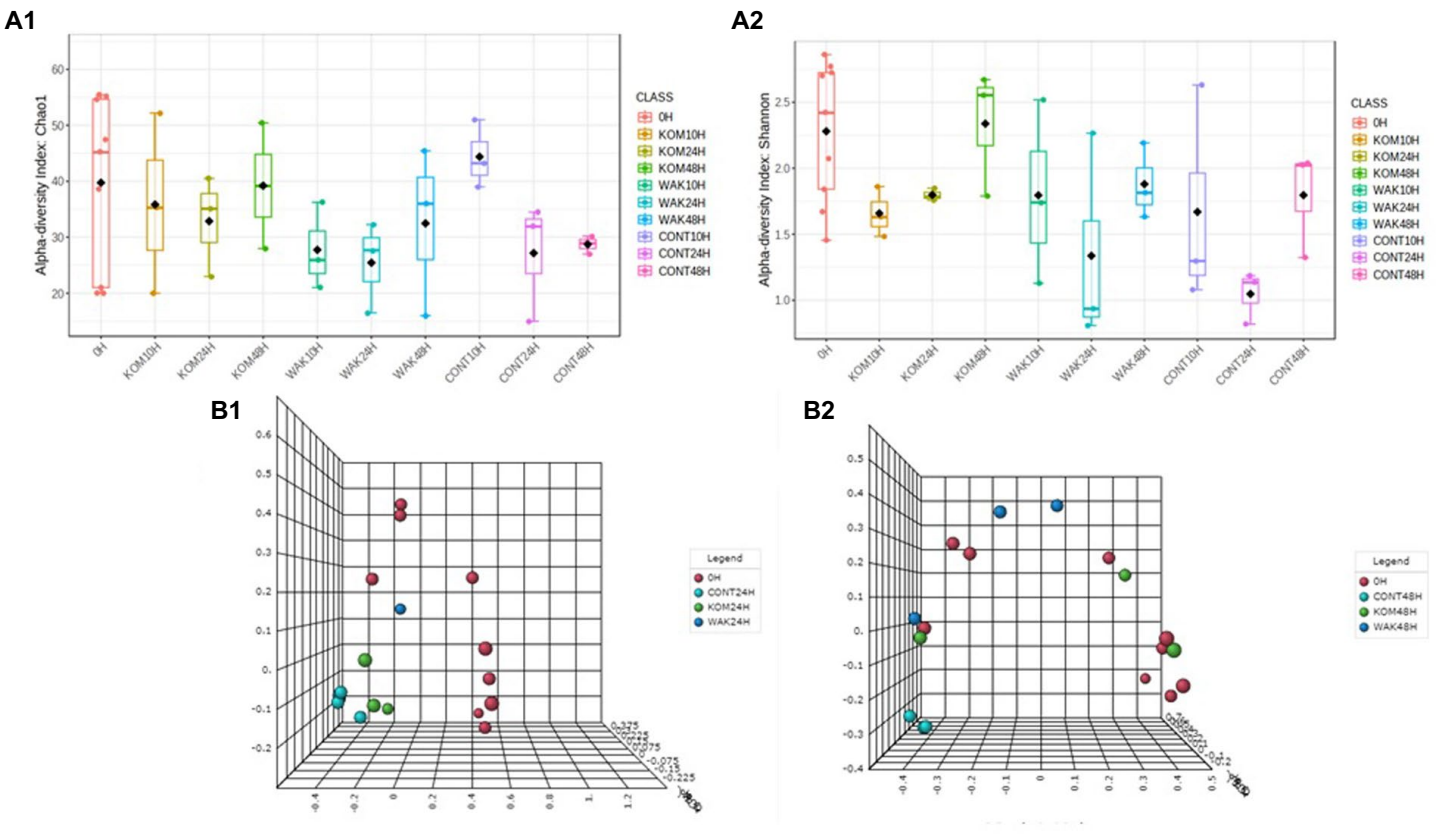
Evolution of the α-diversity, using two indices: Chao (**A**.1) and Shannon (**A**.2), of the operational taxonomic units in the samples and β-diversity, variation of microbial communities between different samples, using Bray–Curtis index at 24 h (**B**.1) and 48 h of each seaweed (**B**.2) compared to baseline (0 h). CONT (negative control, without substrate); KOM (kombu; *S. japonica*); WAK (wakame; *U. pinnatifida*).

Figure 4 shows the *α*-diversity analysis according to the Chao1 and Shannon indices. In terms of the Chao1 index, *α*-diversity decreased at 10 h and 24 h in the two seaweed samples and in the negative control compared to baseline. However, the Chao1 index increased to 48 h for both seaweeds, although to a greater extent for *S. japonica*. In the case of the negative controls, due to the absence of substrate, α-diversity continued to decrease after 48 h. Regarding the Shannon index, in the case of *S. japonica*, an increase was found with respect to the controls at 48 h. No significant differences were found between *U. pinnatifida* and the controls. These data can be compared with those of a study in *H. elongata*, where abundance also increased at 48 h (22).

Figure 4 also shows the *β*-diversity analysis, and two different patterns can be observed at 24 h: (1) 0 h; (2) seaweeds and negative control. At 48 h, three groups can be observed: (1) 0 h, (2) negative control, and (3) seaweeds. This indicates that there are no significant variations in the microbial communities after fermentation of the different substrates. These results are to be expected because fermentation stimulates the growth of GM bacteria but does not modify them.

**Figure 5:**
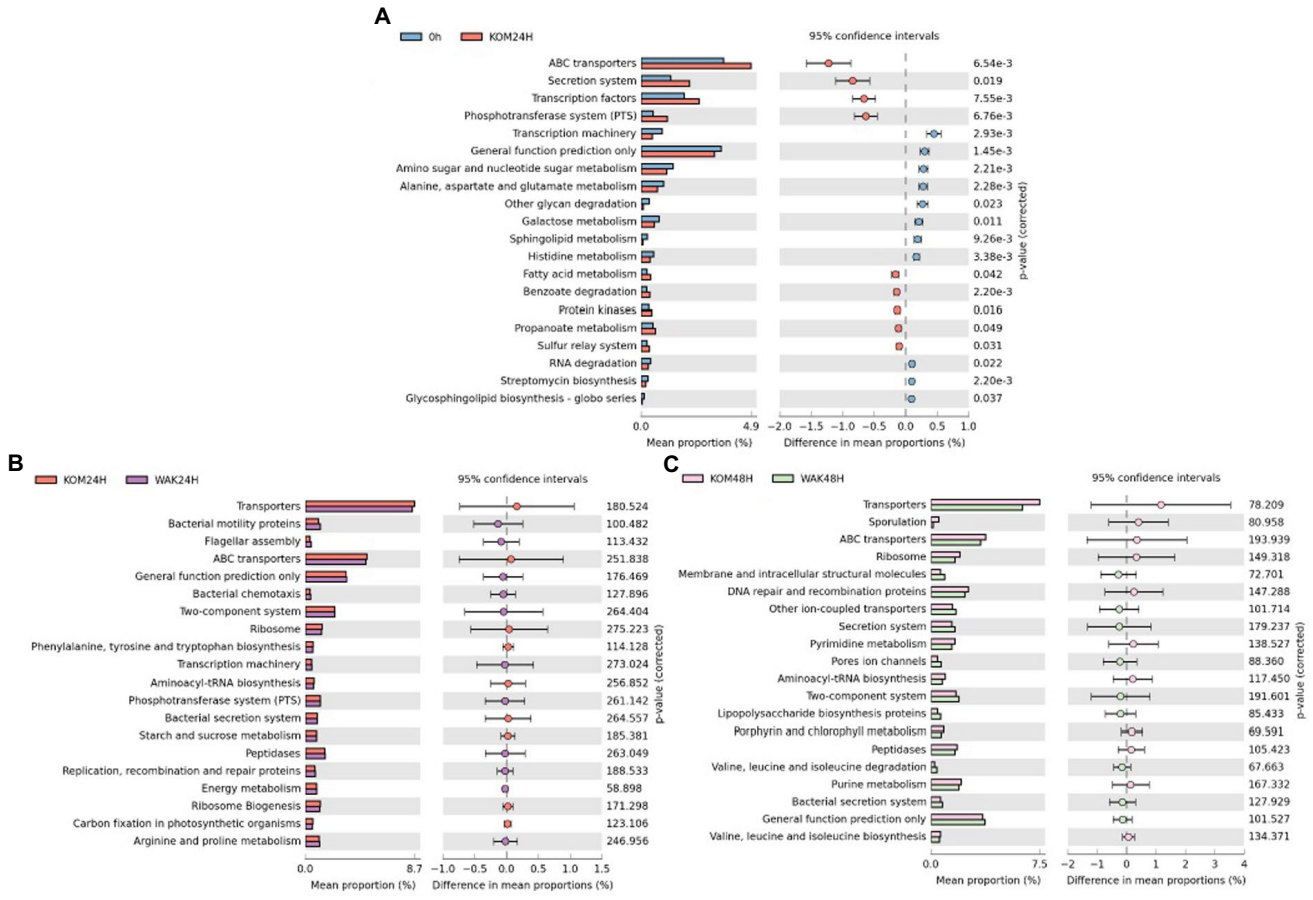
Significant differences of the main 20 metabolic pathways between baseline and *S. japonica* after 24 h fermentation **(A)**; Statistical Analysis Functional Profile of the main 20 metabolic pathways for the samples obtained from the *in vitro* colonic model with *S. japonica* and *U. pinnatifida* at 24 h **(B)** and 48 h **(C)**. The *value of p* obtained for all the metabolic functions (only the 20 more representative in graphics) was higher than 0.05, indicating no significant differences between the times of assay.

Regarding metabolic pathway analysis, Figure 5A shows that the only significant differences were in the treatment with *S. japonica* at 24 h compared to 0 h, in agreement with a previous work carried out on *H. elongata* using inulin (22). In addition, several of the top 20 metabolic pathways coincided in the two studies. No significant differences were observed between the rest of the treatments and times. Figure 5B,C shows that there were no significant differences in the main metabolic pathways between *S. japonica* and *U. pinnatifida* at 24 h and 48 h, respectively.

### 3.3. Short-chain fatty-acid analysis

Table 2 shows the SFCA analysis. As expected, the concentration of total SFCA increased as the hours of fermentation elapsed, although not significantly. The most notable increase occurred in *U. pinnatifida* after 24 h of fermentation, which was also the case in a study on *H. elongata* (22).

**Table 2:** Changes in lactate and short-chain fatty-acid (SCFA) concentrations (mM) in the samples obtained for each substrate after fecal fermentation assays at 0, 10, 24, and 48 h.

		*Saccharina japonica*			*Undaria pinnatifida*			Negative control		
SCFAs	0 h	10 h	24 h	48 h	10 h	24 h	48 h	10 h	24 h	48 h
Lactic	13.41^a^ ± 7.8	2.71^a^ ± 0.59	4.34^a^ ± 1.49	nd	nd	nd	nd	0.09^a^ ± 0.06	0.23^a^ ± 0.17	0.18^a^ ± 0.13
Acetic	3.71^a^ ± 2.13	2.03^a^ ± 0.97	6.05^a^ ± 1.30	13.34^b^ ± 3.41	5.26^a^ ± 1.49	15.45^b^ ± 3.53	16.12^b^ ± 2.65	0.55^a^ ± 0.06	8.2^b^ ± 0.46	9.67^b^ ± 0.33
Propionic	7.09^a^ ± 2.59	6.41^a^ ± 0.24	4.96^a^ ± 1.23	3.23^a^ ± 0.12	5.20^a^ ± 2.34	3.94^a^ ± 1.37	5.36^a^ ± 3.34	1.53^b^ ± 0.05	2.58^b^ ± 0.38	2.34^b^ ± 0.26
Isobutyric	2.20^a^ ± 0.10	10.24^b^ ± 2.40	11.13^b^ ± 5.95	5.35^a,b^ ± 1.67	8.32^b^ ± 3.38	8.15^b^ ± 2.71	0.93^a^ ± 0.77	nd	nd	nd
Butyric	0.55^a^ ± 1.01	0.90^a^ ± 0.80	0.45^a^ ± 0.06	1.02^a^ ± 0.49	7.92^b^ ± 1.23	6.88^b^ ± 2.45	3.54^b^ ± 1.44	0.82^a^ ± 0.32	1.25^a^ ± 0.11	0.79^a^ ± 0.18
Isovaleric	nd	1.58^a^ ± 0.04	2.46^a^ ± 0.19	4.41^a^ ± 2.01	nd	1.07^a^ ± 0.65	1.29^a^ ± 0.42	nd	0.18^a^ ± 0.13	0.54^a^ ± 0.25
Valeric	nd	nd	nd	nd	nd	nd	nd	nd	nd	nd
Total	26.96 ± 13.63	23.87 ± 5.04	29.39 ± 10.22	27.35 ± 7.7	26.7 ± 8.44	35.49 ± 10.71	27.24 ± 8.62	2.99 ± 0.49	12.44 ± 1.25	13.52 ± 1.15

Different lower-case letters indicate significant differences (*p* < 0.05) with time. Different capital letters indicate significant differences (*p* > 0.05) between *Saccharina japonica* and *Undaria pinnatifida* at 24 h and 48 h. Results are expressed as the average ± Standard Error (SE) (*n* = 6). nd, not detected.

Acetic acid increased as the hours of fermentation passed, increasing significantly at 48 h in both seaweeds. These results are similar to those previously obtained by Chen et al. (11), who found an increase in SCFA production as a consequence of seaweed polysaccharide intake. In addition, Bajury et al. (53) found an increase in acetic acid concentrations as a result of seaweed fermentation. Deng et al. (46) also showed an increase in acetic acid by supplementation with dietary fiber extracted from *S. japonica*. Other work carried out with polysaccharides extracted from *U. pinnatifida* also reported an increase in acetic acid production (17). Previous studies suggested that acetic acid could inhibit the growth of enteropathogenic bacteria (54).

For butyric acid, no significant differences were observed between the baseline and *S. japonica* treatments, whereas in the treatment with *U. pinnatifida,* a significant increase was observed at 10 h, followed by a decrease. Jiang et al. (17) showed an increase in butyrate with administration of sulfated polysaccharides extracted from *U. pinnatifida*. This is noteworthy, as it has been shown that butyrate can have positive effects on inflammatory bowel disease, type 1 diabetes mellitus, and nonalcoholic fatty liver disease (55). In addition, butyric acid is an essential source of energy for enterocytes, promoting the recovery and absorption of ions such as calcium, iron, and magnesium and neutralizing ammonium groups generated by deamination of amino acids and other nutrients (56). SCFAs, such as acetic acid, propionic acid, and butyric acid, can inhibit proinflammatory cytokine production and enhance anti-inflammatory effects (17).

## 4. Conclusion

This study evaluated the prebiotic effect of two whole brown seaweeds in an *in vitro* model of the human colon. Both seaweeds were selectively utilized by certain bacteria of the GM, leading to an increase in *Bacteroides* and *Bifidobacterium* species. In terms of metabolic pathways, no differences were found between the two seaweeds, and many of these pathways were also previously associated with inulin, a known prebiotic. This indicates the potential use of both whole seaweeds as prebiotic agents. An increase in the production of short-chain fatty acids was also shown in the fermentation of both seaweeds compared to the negative control. It is important to note that this work has limitations, as it was carried out *in vitro*. However, the results obtained encourage further research into the potential use of both seaweeds as potential prebiotic ingredients in supplements or foods.

## Data availability statement

The original contributions presented in the study are publicly available. This data can be found at: https://www.ncbi.nlm.nih.gov/bioproject/944169.

## Ethics statement

The studies involving human participants were reviewed and approved by Galician Bioethics Committee (trial 270/2018). The patients/participants provided their written informed consent to participate in this study.

## Author contributions

AL-S: methodology, formal analysis, and writing—original draft. AC-C: methodology, formal analysis, writing—original draft, and visualization. AM-P: methodology. LS-L: methodology. JO-V and CMF: conceptualization, investigation, writing—review and editing, visualization, supervision, and project administration. AC: conceptualization, funding acquisition, investigation, writing—review and editing, visualization, supervision, and project administration. All authors contributed to manuscript revision, and read and approved the submitted version.

## Funding

The authors thank the Xunta de Galicia and European Regional Development Funds (FEDER), grant ED431C 2018/05, for covering the cost of the work.

## Conflict of interest

The authors declare that the research was conducted in the absence of any commercial or financial relationships that could be construed as a potential conflict of interest.

## Publisher’s note

All claims expressed in this article are solely those of the authors and do not necessarily represent those of their affiliated organizations, or those of the publisher, the editors and the reviewers. Any product that may be evaluated in this article, or claim that may be made by its manufacturer, is not guaranteed or endorsed by the publisher.

## Footnotes

^1^https://www.microbiomeanalyst.ca/

## References

[1] Cian RE Drago SR De Medina FS Martínez-Augustin O. Proteins and carbohydrates from red seaweeds: evidence for beneficial effects on gut function and microbiota. Mar Drugs. (2015) 13:5358–83. doi: 10.3390/md13085358 26308006 PMC4557026

[2] Mendes MC Navalho S Ferreira A Paulino C Figueiredo D Silva D. Algae as food in Europe: an overview of species diversity and their application. Foods. (2022) 11:1871. doi: 10.3390/foods11131871, PMID: 35804686 PMC9265617

[3] Cronin P Joyce SA PW OT EM OC. Dietary fibre modulates the gut microbiota. Nutrients. (2021) 13:1655. doi: 10.3390/nu13051655, PMID: 34068353 PMC8153313

[4] Stephen AM Champ MMJ Cloran SJ Fleith M van Lieshout L Mejborn H. Dietary fiber in Europe: current state of knowledge on definitions, sources, recommendations, intakes, and relationships to health. Nutr Res Rev. (2017) 30:149–90. doi: 10.3390/nu13010012, PMID: 28676135

[5] Lopez-Santamarina A Miranda JM Mondragon AC Lamas A Cardelle-Cobas A Franco CM. Potential use of marine seaweeds as prebiotics: a review. Molecules. (2020) 25:1004. doi: 10.3390/molecules25041004, PMID: 32102343 PMC7070434

[6] Cosgrove WJ Loucks DP. Water management: current and future challenges and research directions. Water Resour Res. (2015) 51:4823–39. doi: 10.1002/2014WR016869

[7] Charoensiddhi S Conlon MA Vuaran MS Franco CMM Zhang W. The development of seaweed-derived bioactive compounds for use as prebiotics and nutraceuticals using enzyme technologies. Trends Food Sci Technol. (2017) 70:20–33. doi: 10.1016/j.tifs.2017.10.002

[8] Forster J Radulovich R. Seaweed and food security In: Tiwari BK Troy DJ, editors. Seaweed Sustainability: Food and Non-food Applications. London: UK Elsevier Inc (2016). 289–313.

[9] Vaga S Lee S Ji B Andreasson A Talley NJ Agreus L. Compositional and functional differences of the mucosal microbiota along the intestine of healthy individuals. Sci Rep. (2020) 10:14977. doi: 10.1038/s41598-020-71939-2, PMID: 32917913 PMC7486370

[10] Mazziotta C Tognon M Martini F Torreggiani E Rotondo JC. Probiotics mechanism of action on immune cells and beneficial effects on human health. Cells. (2023) 12:184. doi: 10.3390/cells12010184, PMID: 36611977 PMC9818925

[11] Chen L Xu W Chen D Chen G Liu J Zeng X. Digestibility of sulfated polysaccharide from the brown seaweed Ascophyllum nodosum and its effects on the human GM in vitro. Int J Biol Macromol. (2018) 112:1055–61. doi: 10.1016/j.ijbiomac.2018.01.183, PMID: 29425873

[12] Strain CR Collins KC Naughton V McSorley EM Stanton C Smyth TJ. Effects of a polysaccharide-rich extract derived from Irish-sourced Laminaria digitata on the composition and metabolic activity of the human gut microbiota using an in vitro colonic model. Eur J Nutr. (2020) 59:309–25. doi: 10.1007/s00394-019-01909-6, PMID: 30805695 PMC7000515

[13] Vázquez-Rodríguez B Santos-Zea L Heredia-Olea E Acevedo-Pacheco L Santacruz A Gutiérrez-Uribe JA. Effects of phlorotannin and polysaccharide fractions of brown seaweed Silvetia compressa on human gut microbiota composition using an in vitro colonic model. J Funct Foods. (2021) 84:104596. doi: 10.1016/j.jff.2021.104596

[14] Catarino MD Marçal C Bonifácio-Lopes T Campos D Mateus N Silva AMS. Impact of phlorotannin extracts from Fucus vesiculosus on human gut microbiota. Mar Drugs. (2021) 19:375. doi: 10.3390/md19070375, PMID: 34209623 PMC8306378

[15] Roca-Saavedra P Mendez-Vilabrille V Miranda JM Nebot C Cardelle-Cobas A Franco CM. Food additives, contaminants and other minor components: effects on human gut microbiota-a review. J Physiol Biochem. (2018) 74:69–83. doi: 10.1007/s13105-017-0564-2, PMID: 28488210

[16] Shannon E Conlon M Hayes M. Seaweed components as potential modulators of the gut microbiota. Mar Drugs. (2021) 19:358. doi: 10.3390/md19070358, PMID: 34201794 PMC8303941

[17] Jiang P Zheng W Sun X Jiang G Wu S Xu Y. Sulfated polysaccharides from Undaria pinnatifida improved high fat diet-induced metabolic syndrome, gut microbiota dysbiosis and inflammation in BALB/c mice. Int J Biol Macromol. (2021) 167:1587–97. doi: 10.1016/j.ijbiomac.2020.11.116, PMID: 33217459

[18] Wei B Zhang B Du AQ Zhou ZY Lu DZ Zhu ZH. Saccharina japonica fucan suppresses high fat diet-induced obesity and enriches fucoidan-degrading gut bacteria Carbohydr. Polym. (2022) 290:119411. doi: 10.1016/j.carbpol.2022.119411, PMID: 35550744

[19] Association of Official Analytical Chemists (AOAC). Official Methods of Analysis. 17th ed. Gaithersburg, MA: AOAC (2002).

[20] Brodkorb A Egger L Alminger M Alvito P Assunção R Ballance S. INFOGEST static in vitro simulation of gastrointestinal food digestion. Nat Protoc. (2019) 14:991–1014. doi: 10.1038/s41596-018-0119-1, PMID: 30886367

[21] Cardelle-Cobas A Olano A Corzo N Villamiel M Collins M Kolida S. In vitro fermentation of lactulose-derived oligosaccharides by mixed fecal microbiota. J Agric Food Chem. (2012) 60:2024–32. doi: 10.1021/jf203622d, PMID: 22292561

[22] López-Santamarina A Cardelle-Cobas A Mondragón AC Sinisterra-Loaiza L Miranda JM Cepeda A. Evaluation of the potential prebiotic effect of Himanthalia elongata, an Atlantic brown seaweed, in an in vitro model of the human distal colon. Food Res Int. (2022) 156:111156. doi: 10.1016/j.foodres.2022.111156, PMID: 35651022

[23] Gullón B Gullón P Sanz Y Alonso JL Parajó JC. Prebiotic potential of a refined product containing pectic oligosaccharides. LWT. (2011) 44:1687–96. doi: 10.1016/j.lwt.2011.03.006

[24] Bolyen E Rideout JR Dillon MR Bokulich NA Abnet CC Al-Ghalith GA. Reproducible, interactive, scalable and extensible microbiome data science using QIIME 2. Nat Biotechnol. (2019) 37:852–7. doi: 10.1038/s41587-019-0190-3, PMID: 31341288 PMC7015180

[25] Chao A. Nonparametric estimation of the number of classes in a population. Scand J Stat. (1984) 11:265–70. doi: 10.2307/4615964

[26] Kim BR Shin J Guevarra R Lee JH Kim DW Seol KH. Deciphering diversity indices for a better understanding of microbial communities. J Microbiol Biotechnol. (2017) 27:2089–93. doi: 10.4014/jmb.1709.09027, PMID: 29032640

[27] Shannon CE. The mathematical theory of communication. 1963. MD Comput. (1997) 14:306–17. 9230594

[28] Morgan XC Huttenhower C. Chapter 12: human microbiome analysis. PLoS Comput Biol. (2012) 8:e1002808. doi: 10.1371/journal.pcbi.1002808, PMID: 23300406 PMC3531975

[29] Torres MD Flórez-Fernández N Domínguez H. Integral utilization of red seaweed for bioactive production. Mar Drugs. (2019) 17:314. doi: 10.3390/md17060314, PMID: 31142051 PMC6627364

[30] Fernandez-Segovia I Lerma-García MJ Fuentes A Barat JM. Characterization of Spanish powdered seaweeds: composition, antioxidant capacity and technological properties. Food Res Int. (2018) 111:212–9. doi: 10.1016/j.foodres.2018.05.037, PMID: 30007679

[31] Peinado I Girón J Koutsidis G Ames JM. Chemical composition, antioxidant activity and sensory evaluation of five different species of brown edible seaweeds. Food Res Int. (2014) 66:36–44. doi: 10.1016/j.foodres.2014.08.035

[32] Europe Union. Regulation (EC) no 1924/2006 of the European Parliament and of the council of 20 December 2006 on nutrition and health claims made on foods. Off J Eur Union. (2006) L404:9–25.

[33] Siddique MAM Hossain MS Islam MM Rahman M Kibria G. Heavy metals and metalloids in edible seaweeds of Saint Martin’s island, bay of Bengal, and their potential health risks. Mar Pollut Bull. (2022) 181:113866. doi: 10.1016/j.marpolbul.2022.113866, PMID: 35759901

[34] Commission Recommendation (EU) 2018/464 of 19 march 2018 on the monitoring of metals and I in seaweed, halophytes and products based on seaweed. Off J Europ J. (2018) L78:16–8.

[35] Cascais M Monteiro P Pacheco D Cotas J Pereira L Marques JC. Effects of heat treatment processes: health benefits and risks to the consumer. Appl Sci. (2021) 11:8740. doi: 10.3390/app11188740

[36] Commission Regulation (EC) No 1881/2006 of 19 December 2006 setting maximum levels for certain contaminants in foodstuffs. Off J Europ Union. (2006) L364:5–24.

[37] EFSA Panel on Contaminats in the Food Chain (CONTAM). Scientific opinion on lead in food. EFSA J. (2010) 8:1570. doi: 10.2903/j.efsa.2010.1570

[38] EFSA Panel on Contaminats in the Food Chain (CONTAM). Statement on tolerable weekly intake for cadmium. EFSA J. (2011) 9:1975. doi: 10.2903/j.efsa.2011.1975 PMC13090794 42006064

[39] Farias P Marcelino G Santana L de Almeida E Guimarães R Pott A. Minerals in pregnancy and their impact on child growth and development. Molecules. (2021) 25:5630. doi: 10.3390/molecules25235630, PMID: 33265961 PMC7730771

[40] Sizentsov A Sizentsov Y Kvan O Salnokova E Salnokova V. A study on heavy metal sorption properties of intestinal microbiota in vitro. E3S Web Conf. (2019) 79:03021. doi: 10.1051/e3sconf/20197903021

[41] Kumssa DB Joy EJM Ander EL Watts MJ Young S Walker S. Dietary calcium and zinc deficiency risk are decreasing but remain prevalent. Sci Rep. (2015) 5:10974. doi: 10.1038/srep10974, PMID: 26098577 PMC4476434

[42] Kashian S Fathivand AA. Estimated daily intake of Fe, cu, Ca and Zn through common cereals in Tehran. Iran Food Chem. (2015) 176:193–6. doi: 10.1016/j.foodchem.2014.12.021, PMID: 25624223

[43] Europe Union. Council directive 2008/100/CE of 28 October 2008 amending council directive 90/496/EEC on nutrition labelling for foodstuffs as regards recommended daily allowances, energy conversion factors and definitions. Off J Eur Union. (2008) L285:9–12.

[44] Domínguez-González MR Chiocchetti GM Herbello-Hermelo P Vélez D Devesa V Bermejo-Barrera P. Evaluation of I bioavailability in seaweed using in vitro methods. J Agric Food Chem. (2017) 65:8435–42. doi: 10.1021/acs.jafc.7b02151, PMID: 28853868

[45] Seraglio SKT Schulz M Gonzaga LV Fett R Costa ACO. Current status of the gastrointestinal digestion effects on honey: a comprehensive review. Food Chem. (2021) 357:129807. doi: 10.1016/j.foodchem.2021.129807, PMID: 33915465

[46] Deng Z Wu N Wang J Zhang Q. Dietary fibers extracted from Saccharina japonica can improve metabolic syndrome and ameliorate gut microbiota dysbiosis induced by high fat diet. J Funct Foods. (2021) 85:104642. doi: 10.1016/j.jff.2021.104642

[47] Salminen S Collado MC Endo A Hill C Lebeer S Quigley EMM. The international scientific association of probiotics and prebiotics (ISAPP) consensus statement on the definition and scope of postbiotics. Nat Rev Gastroenterol Hepatol. (2021) 18:649–67. doi: 10.1038/s41575-021-00440-6, PMID: 33948025 PMC8387231

[48] Dejean G Tamura K Cabrera A Jain N Pudlo NA Pereira G. Synergy between cell surface glycosidases and glycan-binding proteins dictates the utilization of specific beta (1,3)-glucans by human gut Bacteroides. MBio. (2020) 11:e00095–20. doi: 10.1128/mBio.00095-20 32265336 PMC7157763

[49] De Vadder F Kovatcheva-Datchary P Zitoun C Duchampt A Bäckhed F Mithieux G. Microbiota-produced succinate improves glucose homeostasis via intestinal gluconeogenesis. Cell Metabol. (2016) 24:151–7. doi: 10.1016/j.cmet.2016.06.013, PMID: 27411015

[50] Bui TPN de Vos WM. Next-generation therapeutic bacteria for treatment of obesity, diabetes, and other endocrine diseases. J Clin Endocrinol Metab. (2021) 35:101504. doi: 10.1016/j.beem.2021.101504, PMID: 33785319

[51] Li L Wang Y Yuan J Liu Z Ye C Qin S. Undaria pinnatifida improves obesity-related outcomes in association with gut microbiota and metabolomics modulation in high-fat diet-fed mice. Appl Microbiol Biotechnol. (2020) 104:10217–31. doi: 10.1007/s00253-020-10954-9, PMID: 33074417

[52] Seong H Bae JH Seo JS Kim SA Kim TJ Han NS. Comparative analysis of prebiotic effects of seaweed polysaccharides laminaran, porphyrin, and ulvan using in vitro human fecal fermentation. J Funct Foods. (2019) 57:408–16. doi: 10.1016/J.JFF.2019.04.014

[53] Bajury DM Rawi MH Sazali IH Abdullah A Sardini SR. Prebiotic evaluation of red seaweed (Kappaphycus alvarezii) using in vitro colon model. Int J Food Sci Nutr. (2017) 68:821–8. doi: 10.1016/j.foodres.2022.111156, PMID: 28393631

[54] Fukuda S Toh H Hase K Oshima K Nakanishi Y Yoshimura K. Bifidobacteria can protect from enteropathogenic infection through production of acetate. Nature. (2011) 469:543–7. doi: 10.1038/nature09646 21270894

[55] Liu W Luo X Tang J Mo Q Zhong H Zhang H. A bridge for short-chain fatty acids to affect inflammatory bowel disease, type 1 diabetes, and non-alcoholic fatty liver disease positively: by changing gut barrier. Eur J Nutr. (2021) 60:2317–30. doi: 10.1007/s00394-020-02431-w, PMID: 33180143

[56] Zhang M Wang Y Zhao X Liu C Wang B Zhou J. Mechanistic basis and preliminary practice of butyric acid and butyrate sodium to mitigate gut inflammatory diseases: a comprehensive review. Nut Res Rev. (2021) 95:1–18. doi: 10.1016/j.nutres.2021.08.007, PMID: 34757305

